# Left Main Coronary Artery Spasm in a Hyperthyroid Patient with Suspected Acute Coronary Syndrome

**DOI:** 10.12669/pjms.295.3703

**Published:** 2013

**Authors:** De-zhao Wang, Hong-yu Hu, Qiang Fu, Wei Chen, Xu Hua, Bu-xing Chen

**Affiliations:** 1 De-zhao Wang, Department of Cardiology, Tiantan Hospital, Capital Medical University, Beijing, China.; 2 Hong-yu Hu, Department of Cardiology, Tiantan Hospital, Capital Medical University, Beijing, China.; 3 Qiang Fu, Department of Cardiology, Tiantan Hospital, Capital Medical University, Beijing, China.; 4 Wei Chen, Department of Cardiology, Tiantan Hospital, Capital Medical University, Beijing, China.; 5 Xu Hua, Department of Cardiology, Tiantan Hospital, Capital Medical University, Beijing, China.; 6 Bu-xing Chen, Department of Cardiology, Tiantan Hospital, Capital Medical University, Beijing, China.

**Keywords:** Coronary angiography, Hyperthyroidism, Intravascular ultrasound, Left main coronary artery, Vasospasm

## Abstract

The left main coronary artery (LMCA) vasospasm is rare. We report a suspected acute coronary syndrome patient with hyperthyroidism who had LMCA vasospasm. Coronary angiogram showed 60% stenosis at LMCA. After administering nitroglycerin, re-angiography showed no significant stenosis. Then we evaluated LMCA lesion using intravascular ultrasound (IVUS) showing no significant stenosis. We considered that it was a LMCA vasospasm and may be assosiated with hyperthyroid state. After anti-thyroid and anti-spasm treatment, chest pain subsided. In conclusion, hyperthyroidism induced coronary hypersensitivity may contribute to LMCA vasospasm as seen in this case. IVUS may be useful to identify coronary vasospasm.

## INTRODUCTION

It is well known that coronary artery spasm is an important cause of acute coronary syndrome (ACS). However, a vasospasm of the left main coronary artery (LMCA) is rare. We report the case of a 50-year-old man who was diagnosed with hyperthyroidism and suspected ACS and experienced a LMCA vasospasm when undergoing coronary angiography.

## CASE REPORT

**Fig.1:**
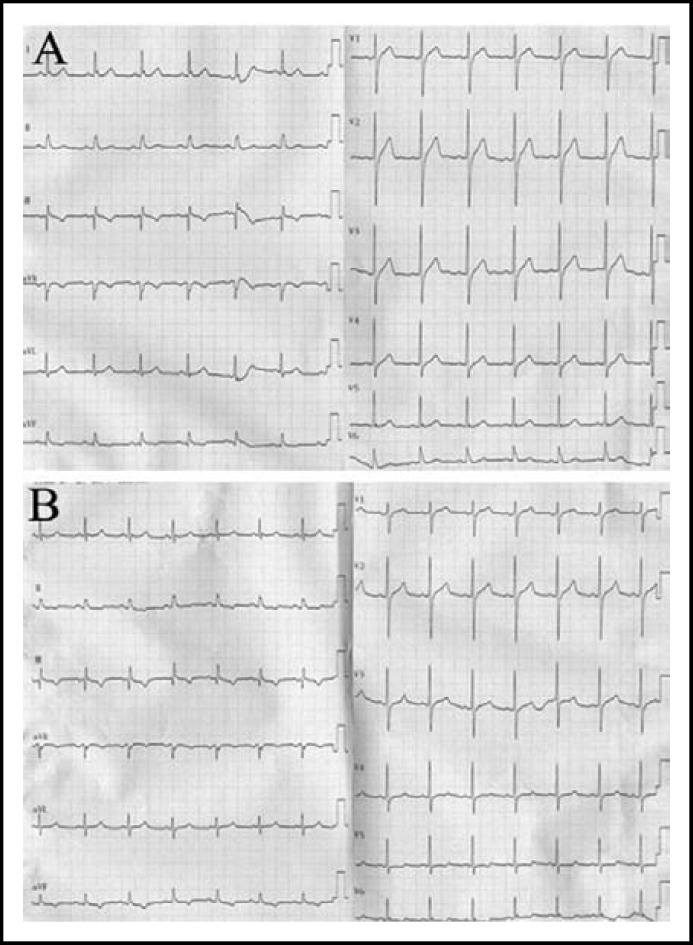
Electrocardiogram (ECG) showing a normalized ST segment deviation while not having episodes of chest pain(A). ECG revealed a 0.1-mm ST segment depression in leads V4-6 (arrows) and up to a 0.05-mm ST segment elevation in the aVR lead (arrows) with the onset of chest pain(B).

We present a 50-year-old man who was admitted to our department complaining of intermittent chest pain. He was a driver by profession and his chest pain was more frequent during driving. He was a past smoker (20 pack years, quitted for 10 years) without any other risk factors of cardiovascular disease. Physical examination showed a blood pressure of 126/70 mmHg, a heart rate of 90 beats/minute, clear lungs and normal heart sounds and no murmurs. The electrocardiogram (ECG) was normal while pain free (Fig.1A). However, ECG revealed 0.1 mm ST segment depression in leads V_5-6 _ and 0.05 mm ST segment elevation in the aVR lead (Fig.1B) during chest pain. Nevertheless, cardiac enzyme profile demonstrated normal creatine phosphokinase (CK), CK-MB and Troponin-I.

**Fig.2:**
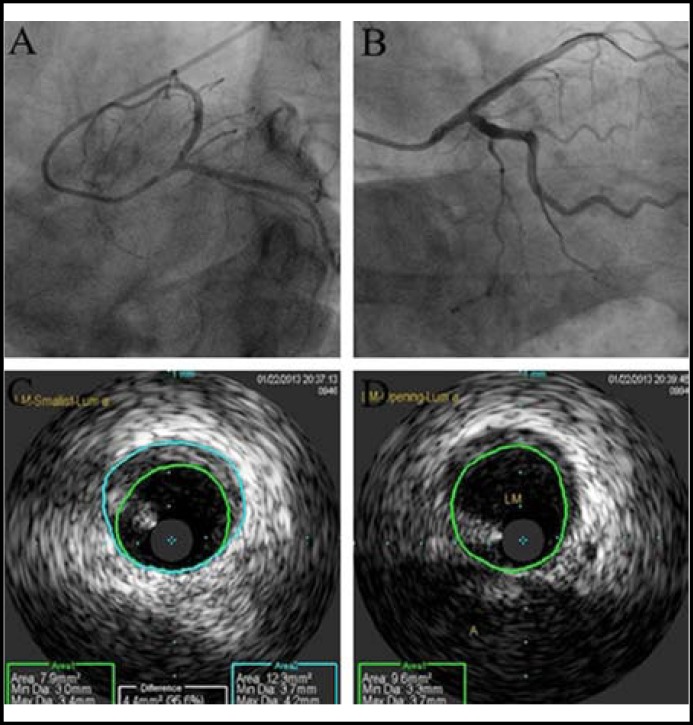
Coronary angiography showing diffuse left main coronary artery vasospasm (A) and resolution of the spasm after administration of intracoronary nitrates (B). Intravascular ultrasound showing a minimal concentric plaque in the left main coronary artery with minimum lumen area of 7.9mm^2^(C) and with maximum lumen area of 9.6mm^2^(D).

Additional laboratory data revealed suppressed thyroid-stimulating hormone (TSH), 0.003 Iu/ml (normal range: 0.35-4.94) and elevation of free thyroxine, 29.71pmol/L (normal range: 9.00-19.04), thyroxine, 212.57nmol/L (normal range: 62.68-150.84), thyroid peroxidase antibody, >1000IU/ml (normal range: 0-12), thyroxine globulin antibody, 238.43IU/ml (normal range: 0-34). Therefore, he was suspected of acute coronary syndrome and hyperthyroidism. Coronary angiogram showed approximately 60% stenosis of the LMCA in its ostium (Fig.2A) and a normal right coronary artery.

The patient complained of chest pain during catheter engagement of LMCA ostium and his systolic blood pressure decreased to 89 mmHg. The catheter was withdrawn immediately with prompt relief of chest pain and rise of systolic blood pressure to 112 mmHg. At that time we thought that coronary intervention might be needed for the lesion of LMCA. Then, the LMCA was engaged with a 6 Fr XB guiding catheter. Two 0.014 inch Balance Middle Weight (BMW) guide wires were inserted into the left anterior descending artery and the left circumflex artery respectively.

However, after administering 100μg of intracoronary nitroglycerin, the re-angiography showed a completely resolved LMCA spasm without residual luminal stenosis (Fig.2B). Intravascular ultrasound (IVUS) showed a minimal concentric plaque in the left main coronary artery with minimum lumen area of 7.9 mm^2 ^(Fig.2C) and with maximum lumen area of 9.6 mm^2 ^(Fig.2D). The diagnosis was confirmed as vasospasm of LMCA. As a result, we abandoned the procedure without stent implantation. Then, the patient was treated with 45 mg of isosorbide dinitrate, 90 mg of diltiazem and 60 mg of methimazol daily. After 7 days of the treatment, the patient’s episodes of chest pain has subsided.

## DISCUSSION

Coronary vasospasm is an important cause of chest pain and may play a crucial role in the pathogenesis of variant angina and ACS. A vasospasm of the left main coronary artery (LMCA) is very rare and the cause of LMCA vasospasm remains unclear.^1^ Kyong et al reported a case of a 35-year-old female of acute myocardial infarction with ST-segment elevation in the aVR lead caused by LMCA vasospasm that was examined on intravascular ultrasound in Korea^2^, but no obvious cause was found. Patel et al reported an atypical case of an African-American woman presenting with acute myocardial infarction due to LMCA spasm, who experienced recurrent angina while hyperthyroid.^3^

The evidence for coronary vasospasm is that CAG showed reversible coronary artery stenosis.^4^ A catheter-induced coronary vasospasm is not uncommon. Thus, in this case, we should distinguish a spontaneous spasm from a catheter-induced spam. In general, a catheter-induced coronary vasospasm appears within 1 mm of the catheter tip and is also resolved by intracoronary nitroglycerin.^5^ In our case, LMCA vasospasm showed irregular and eccentric characteristics and the catheter tip was more than 5 mm from the ostium of the LMCA. Additionally, thyrotoxicosis might induce coronary vasoconstriction despite the fact that predominant effect of thyroxine on the peripheral circulation is to mediate vasodilatation.

Napoli et al studied peripheral arterial reactivity in patients with hyperthyroidism before and after treatment.^6^ They demonstrated though there was nitric oxide mediated basal vasodilatation in untreated hyperthyroidism, the vasoconstriction or response to norepinephrine was significantly enhanced in untreated hyperthyroidism compared to the response in control subjects and in those adequately treated. Consequently, coronary vasospasm in hyperthyroidism might result from an increased sensitivity to norepinephrine or a blunted response to nitric oxide mediated vasodilatation in the coronary arteries.

## CONCLUSION

In conclusion, we describe an interesting case of a hyperthyroid patient who presented with chest pain; had angiographic evidence for vasospasm of the left main coronary artery and had relief of angina after treatment with anti-thyroid drugs. Hyperthyroidism induced coronary hypersensitivity might have contributed to LMCA vasospasm in this case. IVUS may be useful in identifying coronary stenosis or vasospasm, especially for LMCA lesion.
